# Nitrous Oxide and Oxygen

**Published:** 1917-11-03

**Authors:** 


### ANÆSTHETICS IN MILITARY SURGERY.

### NITROUS OXIDE AND OXYGEN.

At the ordinary meeting of the Medical Society of London on Monday, October 29, Captain H. Edmund Boyle, R.A.M.C. (T), anæsthetist to St. Bartholomew's Hospital, introduced an interesting discussion on the use of nitrous oxide and oxygen with re-breathing in military surgery. He said that the anæsthesia produced by the combination of this form with ether, or chloroform and ether when necessary, should appeal to all who had the welfare of their patients at heart, especially on account of its easy induction and the rapid and comfortable recovery from its effects. The method has been largely employed in the United States. His own use of it was due to the personal persuasion of Dr. James Gwathmey, of New York, with whom he had been working at the 1st London General Hospital.

Patients were given a preliminary injection of morphia, atropine, and scopolamine half an hour before the operation, though in busy practice some patients were operated upon before that interval had elapsed, while for others the time was longer. The proportions which the author used were morphia tartrate, $\frac{1}{6}$ gr.; atropine sulphate, $\frac{1}{120}$ gr.; scopolamine $\frac{1}{100}$ gr. This is so made up that five minims constitute a dose. When made in bulk, if the preparation was not used at once it became flocculent, to remedy which one drop of pure carbolic was added to every 4 oz. of the mixture. The apparatus used—Gwathmey's—consists of four cylinders, *i.e.*, two for $N_2O$, two for O, the supply of which was regulated by two graduated taps permitting the gases to flow through the glass bottle containing water, which gives visual evidence of the quantity of each being inhaled. The patient may receive the gases *via* the re-breathing bag, or *via* a bottle containing ether, or one with chloroform and ether. At the top of the face-piece is a valve which allows of intake and output of air and mixed gases, but in practice this valve is only used for those who will not stand prolonged re-breathing and are liable to vomit during the period of anæsthesia.

During induction the room should be thoroughly quiet, a point not sufficiently attended to as a rule. It is especially necessary as the patient's hearing at this crisis in his life is particularly acute. The patient should lie absolutely flat, in Gwathmey's opinion, but Captain Boyle's practice is to have the head slightly raised during induction, gently lowering it to the horizontal when anæsthesia is complete. It is well to insert a small dental prop between the teeth before the induction, though this does not seem to be so necessary as when using gas and ether. It is always needed when breathing is not taking place easily through the nose.

The bag is three-parts filled with $N_2O$ and O in the proportion of 4 to 1; *i.e.*, oxygen bubbles through one hole, nitrous oxide through four. The face-piece is accurately put on, and the gas-valve turned on. If the face-piece does not fit well, there will be excitement and struggling, and the anæsthesia will be hampered. The face-piece valve is kept open during a few breaths, then closed, so that re-breathing takes place almost from the beginning. The supply of $N_2O$ is now increased until it is flowing in freely through all the apertures, and even through the tube, so as to shorten the period of induction. The breathing soon becomes deeper, and in three to four minutes it has assumed the automatic rhythm which speaks of anæsthesia. Strict watch must be kept on the patient's colour, any degree of cyanosis being remedied by adding only a little oxygen. If the patient begins to retch or vomit, there is probably too much re-breathing, and the face-piece valve should be opened, and the supply of both oxygen and nitrous oxide increased. To deepen the degree of anæsthesia the pressure in the bag must be so increased that when an expiration takes place the bag becomes taut. Captain Boyle agrees with Page that it is better to deepen anæsthesia by giving ether.

Over-dosage is indicated by noisy and stertorous breathing, followed by muscular twitching and accompanied by cyanosis. The remedy is to give more oxygen. Anxious movements of the gas-taps lead to bad anæsthesia. The importance of the open and free air-way must never be forgotten. Sometimes it is necessary to add either C.E. mixture or ether to the gas and oxygen when extra relaxation of muscles is required, or when nerves are being severed, as in amputations. Captain Boyle would not at present give a definite opinion on the relative values of ether, or chloroform and ether, with this method; but he had only added chloroform for an exceptionally burly or alcoholic patient.

He fully agrees with Gwathmey's remarks on re-breathing in his book on anæsthesia. That authority says that for an even anæsthesia and as an aid to the avoidance of surgical shock a certain amount of re-breathing is of benefit. The rate of flow of the gases should be such that from a quarter to a half of the volume of each respiration is of freshly added gas mixture. Too much re-breathing is apt to be followed by post-operative discomfort. Vomiting during anæsthesia often indicates excessive re-breathing for the particular patient. An increase of the volume of the gas mixture often clears up the symptoms. The patient must always be pink; anæsthesia accompanied by cyanosis is dangerous.

If there be an overdose of nitrous oxide in the presence of sufficient oxygen to keep the patient pink, there is stertorous respiration and an excessive secretion of mucus. Unless the percentage of nitrous oxide be then decreased, the patient's face and hands assume a death-like pallor, the facial reflexes are quite lost, respirations become shallow, and probably the blood pressure falls. The treatment for overdose is rapid removal of the mask and

**ANÆSTHETICS IN MILITARY SURGERY**—(*continued*).

the commencement of artificial respiration and the administration of oxygen, always remembering to keep the air-way clear.

The post-operative condition of patients so anæsthetised is strikingly good. They are conscious within two or three minutes of the removal of the anæsthetic, and in the majority of cases are not sick. Of 200 consecutive cases at the 1st London General Hospital, thirteen were sick once, four twice, and three had prolonged sickness. In a fair proportion there was twenty minutes of nausea; occasionally some headache; but beyond this, recovery was perfect. In most of his 200 consecutive cases at the 1st London General Hospital there was no appreciable rise of temperature after the operation.

Of 711 cases in which the method was used, there were five cases of bronchitis, and of 550 cases 38 per cent. had been given either ether or C.E. mixture. There were no fatal cases. Only 1.5 per cent. had had bad vomiting.

The author does not contend that this method is suitable for all cases. He has found it unsuitable for (1) cases in which absolute relaxation is required all the time; (2) some abdominal cases, such as gall-bladder operations. For tonsils and adenoids Gwathmey has perfected a special technique.

The longest time during which anæthesia has been maintained by this method in the hands of the author is 2¾ hours. Capt. Boyle is of opinion that this method is in advance of the older ones, and would probably be greatly appreciated in hospitals at the Front, particularly as it would lessen the work of orderlies and nurses, owing to the lack of post-operative shock. But it should never be entrusted to the inexpert.

